# Supplementary material for: Impact of Hypermannosylation on the Structure and Functionality of the ER and the Golgi Complex
Source: Biomedicines. 2023 Jan 6;11(1):146. doi: 10.3390/biomedicines11010146 (PMC9856158; doi:10.3390/biomedicines11010146)
Supplement: Supplementary file 1 [file biomedicines-11-00146-s001.zip › biomedicines-1977902-supplementary.pdf]

biomedicines

Supplemental Material

Impact of Hypermannosylation on the Structure and Functionality of the ER and the Golgi Complex

Patricia Franzka [1,*], Svenja Caren Schüler [2], Takfarinas Kentache [3], Robert Storm [4], Andrea Bock [1], Istvan Katona [5], Joachim Weis [5], Katrin Buder [2], Christoph Kaether [2] and Christian A. Hübner [1,*]

[1] Institute of Human Genetics, University Hospital Jena, 07747 Jena, Germany
[2] Leibniz-Institute on Aging—Fritz-Lipmann-Institute, 07745 Jena, Germany
[3] De Duve Institute, UCLouvain, BE-1200 Woluwe-Saint-Lambert, Belgium
[4] Molecular Devices, 81377 München, Germany
[5] Institute of Neuropathology, RWTH Aachen University Hospital, 52074 Aachen, Germany
* Correspondence: patricia.franzka@med.uni-jena.de (P.F.); christian.huebner@med.uni-jena.de (C.A.H.)

Supplementary Materials: The following supporting information can be downloaded at: www.mdpi.com/xxx/s1. Figure S1: Quantitative changes of proteins necessary for ER and Golgi organization and function in GMPPA KO skeletal muscle and brain compared to WT upon Con A pulldown; S2: Significantly altered ER- and Golgi-resident proteins in GMPPA KO mice upon Con A pulldown; S3: Protein interaction network of Con A enriched proteins necessary for ER and Golgi function and organization; Figure S4: Example for ComDet analysis in ImageJ; Figure S5: Mannose levels are increased in the supernatant of GMPPA KO MEFs; Figure S6: Increased mannosylation in WT MEFs treated with mannose; Table S1: ER- and Golgi-resident proteins.

Raw figures can be found at: https://cloud.med.uni-jena.de/index.php/s/E775K6h9zsGOzr1

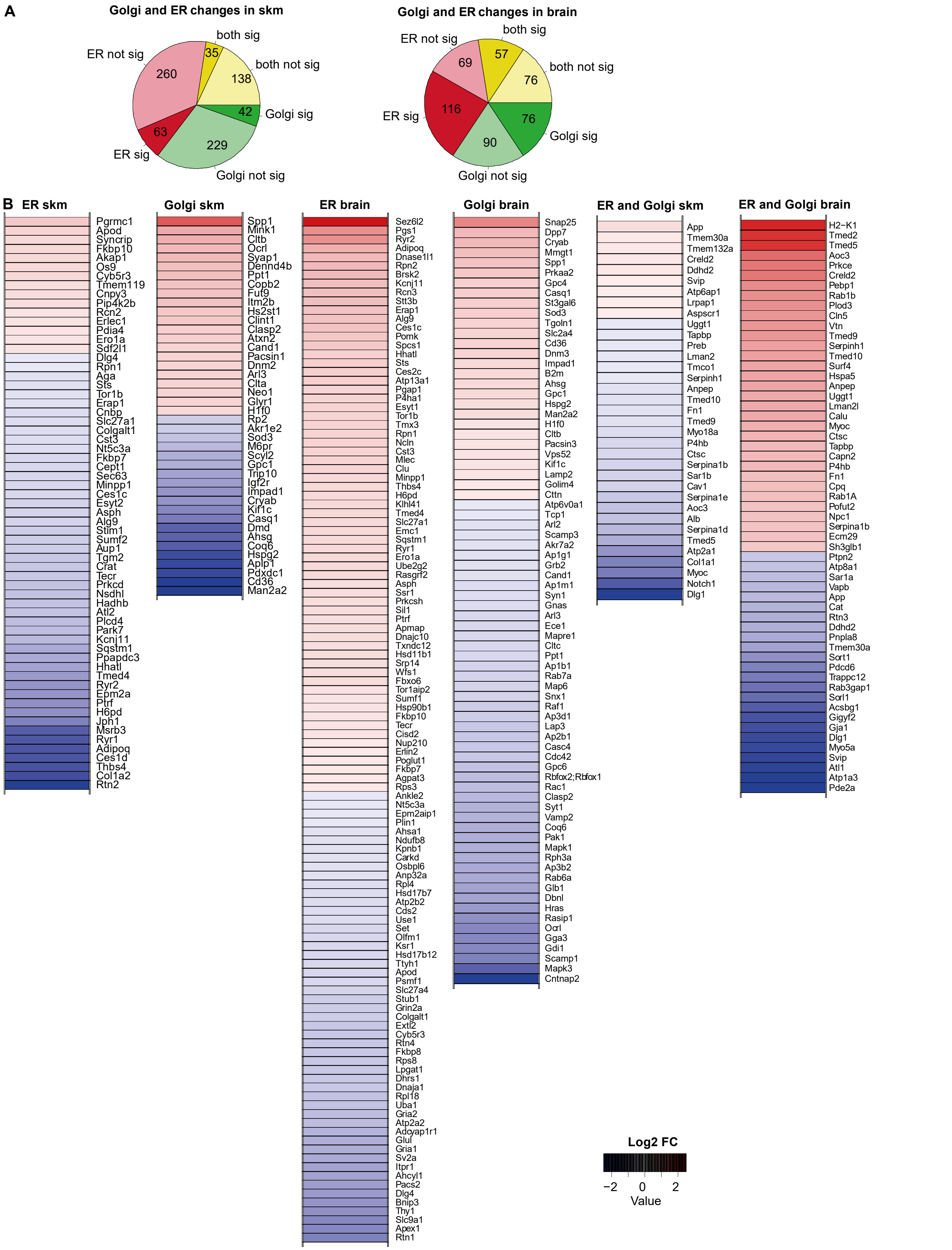

Supplementary Figure S1. Quantitative changes of proteins necessary for ER and Golgi organization and function in GMPPA KO skeletal muscle and brain compared to WT upon Con A pulldown. A) Pie chart for altered ER- and Golgi-resident proteins

in skeletal muscle (skm) and brain of GMPPA KO mice after Con A enrichment. Significant (sig) values show a q-value below 0.05 and an absolute fold change (Log2 FC) above 0.5. Proteins significantly up-regulated between WT and GMPPA KO are shown in red and those down-regulated in blue (n = 3 mice per group). **B)** Depicted are heatmaps for all significantly altered (q-value below 0.05 and fold change above 0.5 or below -0.5) proteins resident in ER and/or Golgi apparatus for skm and brain tissue of GMPPA KO mice after Con A pulldown (n=3 mice per group).

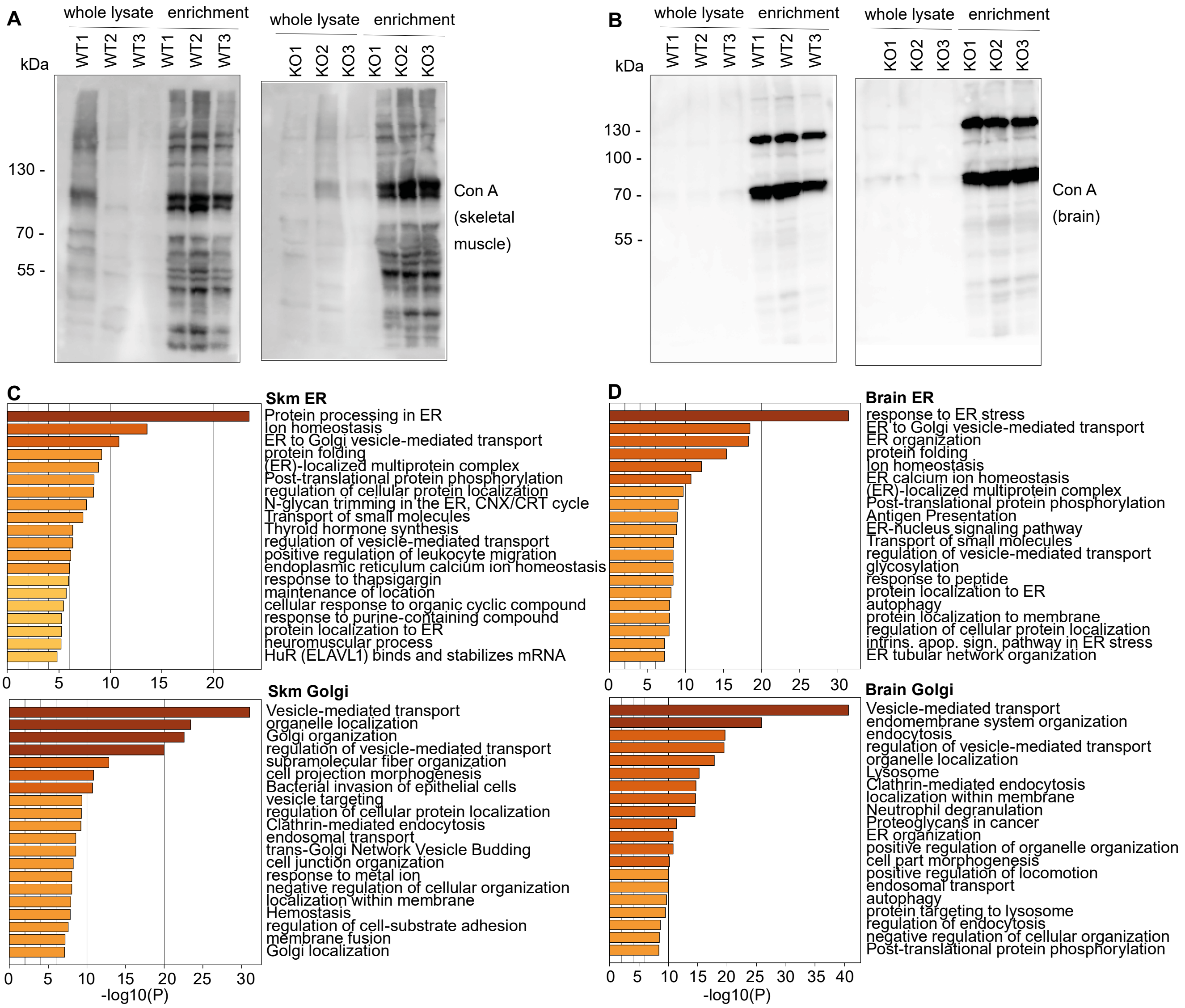

Supplementary Figure S2. Affected pathways upon Con A pulldown in GMPPA KO mice. A-B) Western Blot membranes probed for Con A for whole tissue samples as well as afer Con A enrichment. **A)** Membranes showing skeletal muscle samples. **B)** Membranes showing brain samples. **C-D)** Metascape analysis for involved biological processes for all in the Con A pulldown identified proteins with a q-value below 0.05 for: **C)** ER-resident and Golgi-resident proteins in skm. **D)** ER-resident and Golgi-resident proteins in brain.

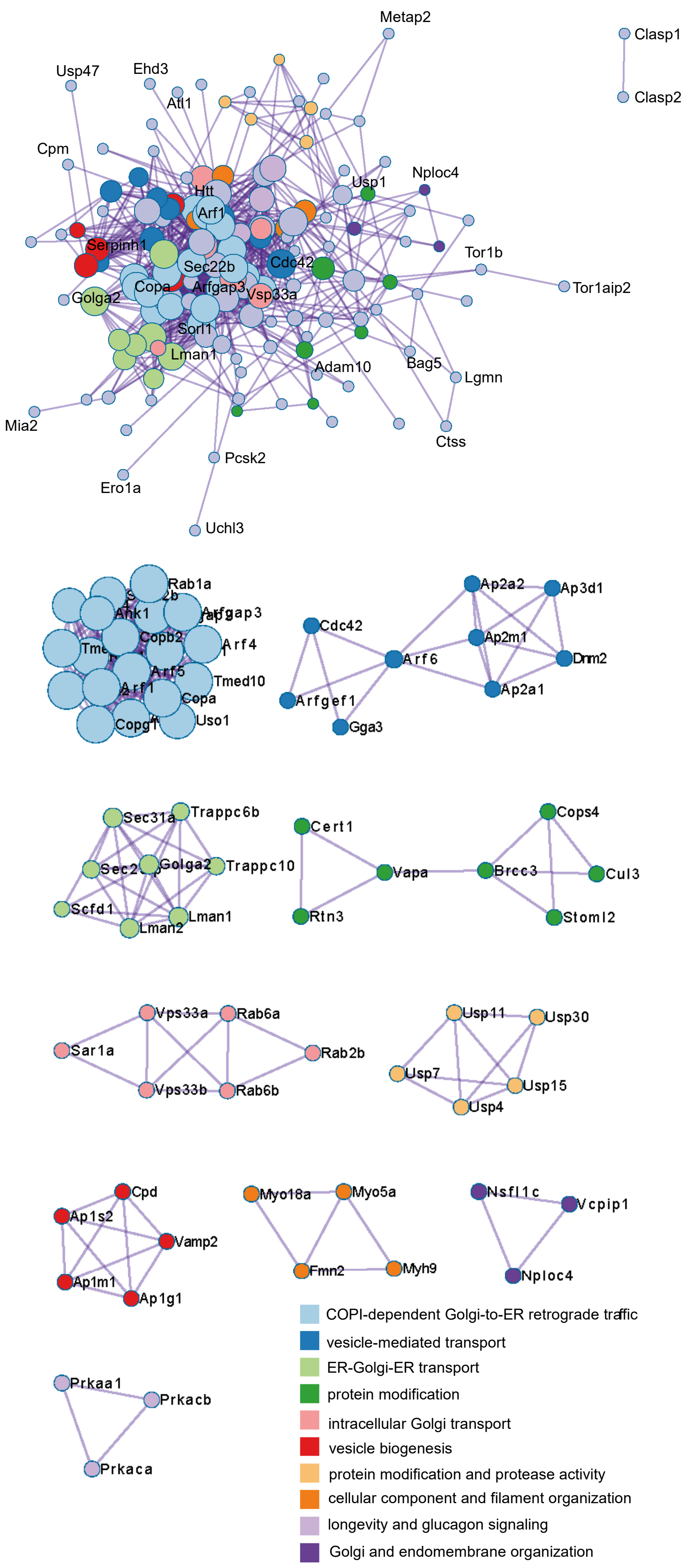

Metap2
Clasp1
Clasp2
Usp47
Ehd3
Atl1
Cpm
Htt
Arf1
Nploc4
Usp1
Serpinh1
Sec22b
Cdc42
Tor1b
Tor1aip2
Copa
Arfgap3
Vsp33a
Golga2
Sorl1
Lman1
Adam10
Bag5
Lgmn
Mia2
Ctss
Pcsk2
Ero1a
Uchl3
Rab1a
Ank1
Copb2
Arf4
Arf5
Tmed10
Copg1
Uso1
Ap2a2
Ap3d1
Cdc42
Ap2m1
Arf6
Dnm2
Arfgef1
Ap2a1
Gga3
Sec31a
Trappc6b
Golga2
Trappc10
Scfd1
Lman1
Lman2
Cert1
Vapa
Rtn3
Cops4
Brcc3
Cul3
Stoml2
Vps33a
Rab6a
Sar1a
Rab2b
Vps33b
Rab6b
Usp11
Usp30
Usp7
Usp15
Usp4
Cpd
Ap1s2
Vamp2
Ap1m1
Ap1g1
Myo18a
Myo5a
Fmn2
Myh9
Nsfl1c
Vcpip1
Nploc4
Prkaa1
Prkacb
Prkaca
COPI-dependent Golgi-to-ER retrograde traffic
vesicle-mediated transport
ER-Golgi-ER transport
protein modification
intracellular Golgi transport
vesicle biogenesis
protein modification and protease activity
cellular component and filament organization
longevity and glucagon signaling
Golgi and endomembrane organization

Supplementary Figure S3. Protein interaction network of Con A enriched proteins necessary for ER and Golgi function and organization. Depicted are identified protein interactions via Metascape analysis of proteins from skeletal muscle and brain selected for the pathways shown in Figure 2 and 3.

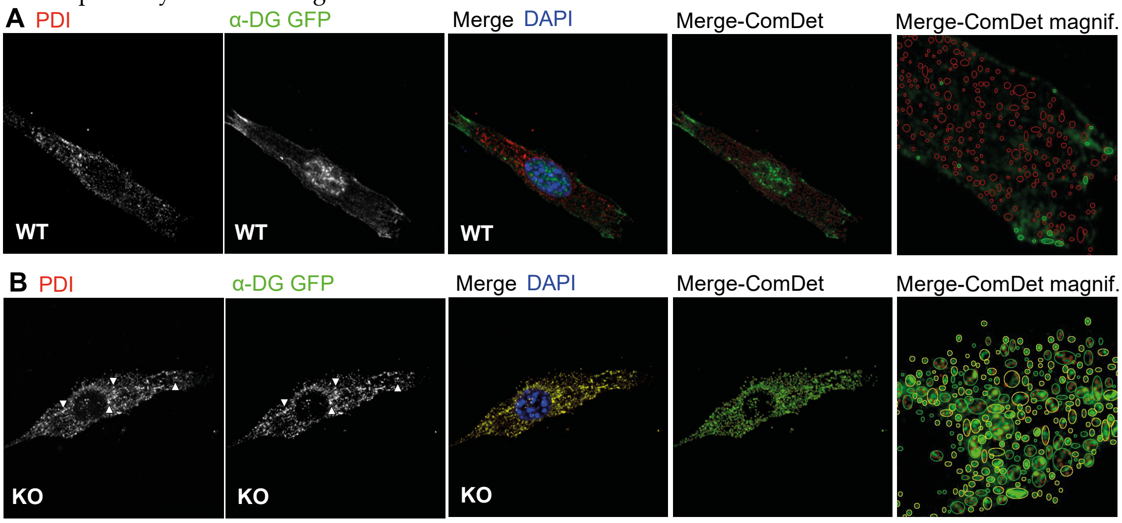

Supplementary Figure S4. Example for ComDet analysis in ImageJ. A-B) Representative images (displayed in Figure 4) and the according ComDet plugin analysis with magnifications of **A)** WT and **B)** GMPPA KO MEFs transfected with α-DG-GFP and stained for PDI (scale bars: 8 µm). White arrows indicate colocalized structures. Green circles: α-DG-GFP detected structures. Red circles: Detected structures for PDI. Yellow circles: detected structures with colocalization of PDI and α-DG-GFP.

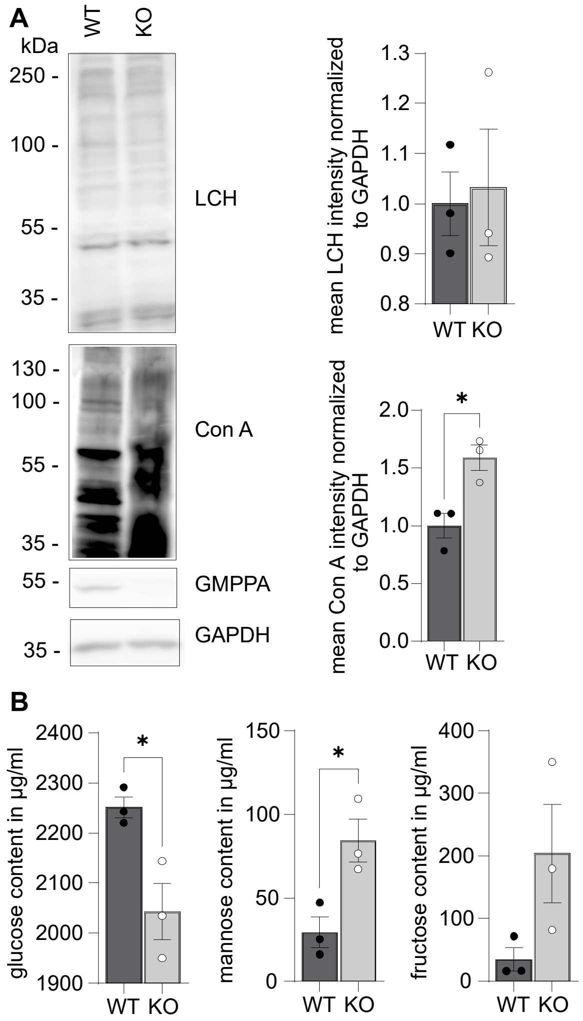

Supplementary Figure S5. Mannose levels are increased in the supernatant of GMPPA KO MEFs. A) Representative Western Blot images for the lens culinaris lectin (LCH) and the concanavalin A lectin (Con A) with according analysis (n=3 experiments per group).

GMPPA served for genotype verification and GAPDH as loading control (n=3 experiments per group). **B)** Mannose, glucose and fructose detection in the supernatant of WT and GMPPA KO MEFs (n=3 experiments per group). Two-tailed Student´s T-Test.

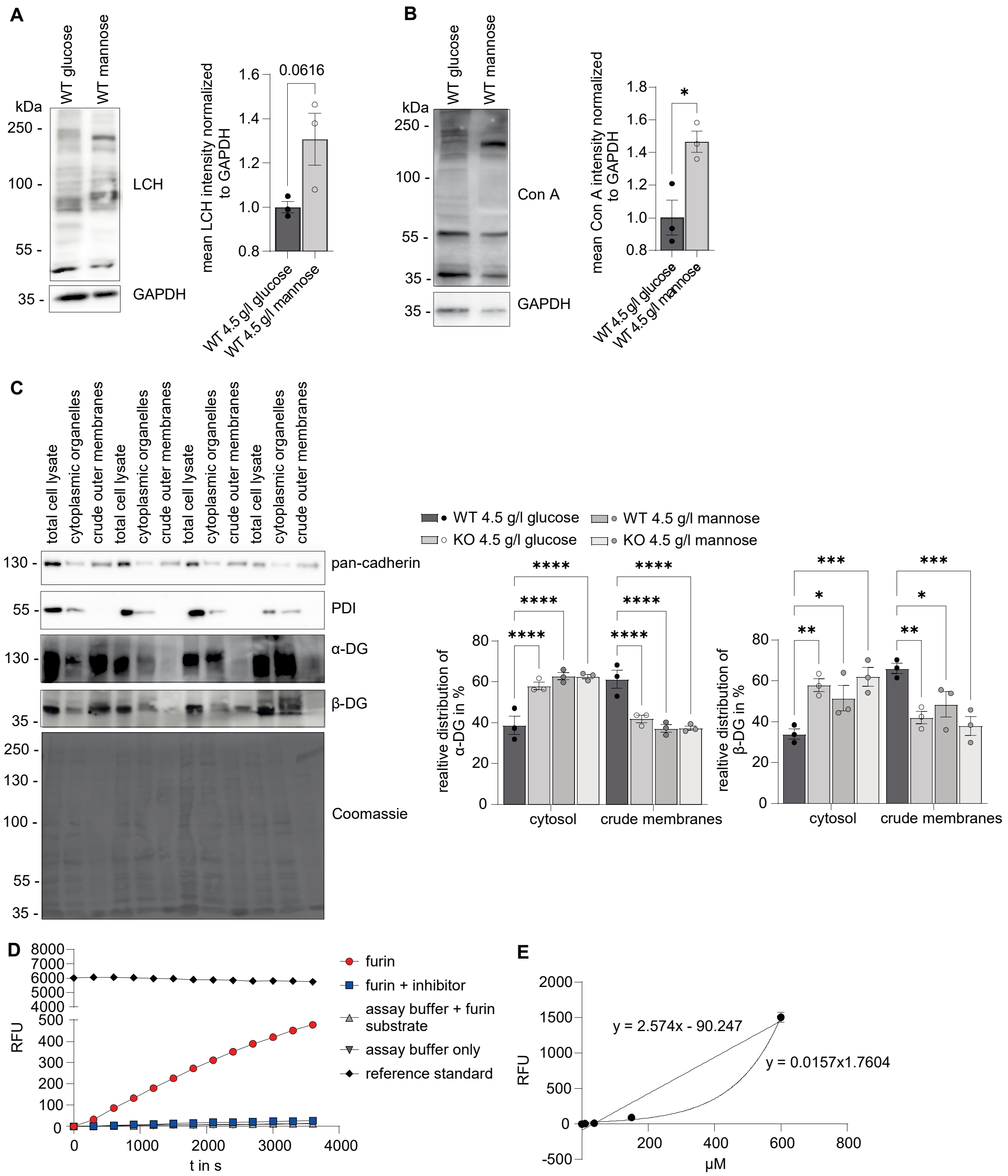

Supplementary Figure S6. Increased mannosylation in WT MEFs treated with mannose. A) Representative Western Blot analysis with the lens culinaris lectin (LCH) with according analysis (n=3 experiments per group). GAPDH served as loading control. Two-tailed Student´s T-Test. **B)** Representative Western Blot membranes with the concanavalin A lectin (Con A) with according analysis (n=3 experiments per group). GAPDH served as loading control. Two-tailed Student´s T-Test. **C)** Western Blot analysis of subcellular fractionation with according analysis of α- and β-DG distribution (n=3 experiments per group). 2-way-ANOVA with Bonferroni post-hoc analysis. Coomassie blue staining served as loading control, pan-cadherin served as a marker of the plasma membrane and PDI served as a marker for the cytoplasmic organelle fraction. WT and GMPPA KO cells were transfected with a dystroglycan construct, treated with either glucose (4.5 g/l) or mannose (4.5 g/l) and then fractionated. **D-E)** Furin activity reference curves. **D)** Verification of the furin activity assay: Recombinant furin shows an increase in relative fluorescence units (RFU) with increasing time, which is absent in substrate controls. A fluorescence standard that resembles cleaved furin substrate showed a slight decrease in RFU over time. **E)** Dilution series of recombinant furin enzyme after incubation with furin substrate for 40 min.
